# Supplementary material for: Genome-wide identification of 2-oxoglutarate and Fe (II)-dependent dioxygenase family genes and their expression profiling under drought and salt stress in potato
Source: PeerJ. 2023 Nov 20;11:e16449. doi: 10.7717/peerj.16449 (PMC10666615; doi:10.7717/peerj.16449)
Supplement: Supplemental Information 1 [file peerj-11-16449-s001.docx]

| **Organism** | **Protein ID** |
| --- | --- |
| *Arabidopsis thaliana* | AAL14647.1 |
| *Arabidopsis thaliana* | AAL14646.1 |
| *Arabidopsis thaliana* | AAG52431.1 |
| *Arabidopsis thaliana* | AAC79100.1 |
| *Arabidopsis thaliana* | AAL14667.1 |
| *Arabidopsis thaliana* | AAL14665.1 |
| *Arabidopsis thaliana* | AAL14644.1 |
| *Arabidopsis thaliana* | AAL14682.1 |
| *Arabidopsis thaliana* | AAC79095.1 |
| *Arabidopsis thaliana* | AAL14643.1 |
| *Arabidopsis thaliana* | AAL14699.1 |
| *Arabidopsis thaliana* | AAL14695.1 |
| *Arabidopsis thaliana* | AAL14692.1 |
| *Arabidopsis thaliana* | AAL14689.1 |
| *Arabidopsis thaliana* | AAL14672.1 |
| *Arabidopsis thaliana* | AAL14670.1 |
| *Arabidopsis thaliana* | AAL14669.1 |
| *Arabidopsis thaliana* | AAL14694.1 |
| *Arabidopsis thaliana* | AAL14688.1 |
| *Arabidopsis thaliana* | AAL14686.1 |
| *Arabidopsis thaliana* | AAL14680.1 |
| *Nicotiana tabacum* | BAA32156.1 |
| *Nicotiana tabacum* | CAA58232.1 |
| *Nicotiana tabacum* | NP_001312327.1 |
| *Nicotiana tabacum* | NP_001312109.1 |
| *Nicotiana tabacum* | NP_001312238.1 |
| *Nicotiana tabacum* | XP_016483159.1 |
| *Nicotiana tabacum* | XP_016453365.1 |
| *Nicotiana tabacum* | XP_016440664.1 |
| *Manihot esculenta* | XP_021599097.1 |
| *Manihot esculenta* | XP_021607152.1 |
| *Manihot esculenta* | XP_021630561.1 |
| *Manihot esculenta* | XP_021606350.1 |
| *Manihot esculenta* | XP_021598609.1 |
| *Manihot esculenta* | XP_021631530.1 |
| *Manihot esculenta* | XP_021629204.1 |
| *Manihot esculenta* | XP_021624634.1 |
| *Manihot esculenta* | XP_021630581.1 |
| *Manihot esculenta* | AAX84675.1 |
| *Manihot esculenta* | XP_021605532.1 |
| *Manihot esculenta* | XP_043806881.1 |
| *Solanum chacoense* | AAC95363.1 |
| *Capsicum annum* | XP_016545848.1 |
| *Capsicum annum* | XP_016560079.1 |
| *Capsicum annum* | XP_047260935.1 |
| *Capsicum annum* | XP_016559561.2 |
| *Capsicum annum* | KAF3676330.1 |
| *Capsicum annum* | XP_016546723.1 |
| *Capsicum annum* | XP_016564734.1 |
| *Capsicum annum* | XP_016572277.2 |
| *Capsicum annum* | KAF3666323.1 |
| *Capsicum annum* | KAF3641865.1 |
| *Capsicum annum* | KAF3654823.1 |
| *Capsicum annum* | KAF3645188.1 |
| *Capsicum annum* | XP_016568125.1 |
| *Solanum melongena* | QOE88789.1 |
| *Solanum melongena* | ANN02873.1 |
| *Solanum melongena* | UXQ89721.1 |
| *Solanum melongena* | CAA54557.1 |
| *Solanum melongena* | BAF03078.1 |
| *Nicotiana tabacum* | BAA32156.1 |
| *Nicotiana tabacum* | CAA58232.1 |
| *Nicotiana tabacum* | NP_001312327.1 |
| *Nicotiana tabacum* | NP_001312109.1 |
| *Nicotiana tabacum* | NP_001312238.1 |
| *Nicotiana tabacum* | XP_016483159.1 |
| *Nicotiana tabacum* | XP_016453365.1 |
| *Nicotiana tabacum* | XP_016440664.1 |
| *Solanum lycopersicum* | XP_004247707.1 |
| *Solanum lycopersicum* | XP_010321064.1 |
| *Solanum lycopersicum* | XP_010315984.1 |
| *Solanum lycopersicum* | XP_004248563.1 |
| *Solanum lycopersicum* | NP_001234747.2 |
| *Solanum lycopersicum* | XP_019071408.1 |
| *Solanum lycopersicum* | XP_004248394.2 |
| *Solanum lycopersicum* | XP_004232746.2 |
| *Solanum lycopersicum* | XP_004242519.1 |
| *Solanum lycopersicum* | XP_004233006.1 |
| *Solanum lycopersicum* | XP_004252111.1 |
| *Solanum lycopersicum* | XP_004230829.1 |
| *Solanum lycopersicum* | NP_001234579.1 |
| *Solanum lycopersicum* | NP_001234070.1 |
| *Solanum lycopersicum* | XP_004246497.1 |
| *Solanum lycopersicum* | NP_001233848.2 |
| *Solanum lycopersicum* | NP_001234363.1 |
| *Solanum lycopersicum* | XP_004247234.1 |
| *Solanum lycopersicum* | NP_001234624.1 |
| *Solanum lycopersicum* | XP_004250329.1 |
| *Solanum lycopersicum* | NP_001316412.1 |
| *Solanum lycopersicum* | NP_001361323.1 |
| *Solanum lycopersicum* | XP_004250780.1 |
