# Supplementary material for: Genome-wide identification of 2-oxoglutarate and Fe (II)-dependent dioxygenase family genes and their expression profiling under drought and salt stress in potato
Source: PeerJ. 2023 Nov 20;11:e16449. doi: 10.7717/peerj.16449 (PMC10666615; doi:10.7717/peerj.16449)
Supplement: Supplemental Information 2 [file peerj-11-16449-s002.docx]

| **Transcript IDs** | **Chromosome** | **Amino Acid Length** | **Nomenclature** | **MW(daltons)** | **pI** | **Subcellular Localisation** | **GRAVY** |
| --- | --- | --- | --- | --- | --- | --- | --- |
| [Soltu.DM.01G002180.1](http://spuddb.uga.edu/cgi-bin/annotation_report.cgi?orf=Soltu.DM.01G002180.1) | 1 | 276 | St2ODD1 | 31555.84 | 5.37 | Extracellular | -3.7 |
| [Soltu.DM.01G018680.1](http://spuddb.uga.edu/cgi-bin/annotation_report.cgi?orf=Soltu.DM.01G018680.1) | 1 | 326 | St2ODD14 | 36530.55 | 6.46 | Cytoplasmic | -0.32 |
| [Soltu.DM.01G030770.1](http://spuddb.uga.edu/cgi-bin/annotation_report.cgi?orf=Soltu.DM.01G030770.1) | 1 | 281 | St2ODD18 | 31669.03 | 5.15 | Extracellular | -0.23 |
| [Soltu.DM.01G048570.1](http://spuddb.uga.edu/cgi-bin/annotation_report.cgi?orf=Soltu.DM.01G048570.1) | 1 | 354 | St2ODD27 | 40966.14 | 5.27 | Extracellular | -0.54 |
| [Soltu.DM.01G025170.1](http://spuddb.uga.edu/cgi-bin/annotation_report.cgi?orf=Soltu.DM.01G025170.1) | 1 | 346 | St2ODD17 | 38741.76 | 6.08 | Extracellular | -0.3 |
| [Soltu.DM.01G010800.1](http://spuddb.uga.edu/cgi-bin/annotation_report.cgi?orf=Soltu.DM.01G010800.1) | 1 | 349 | St2ODD10 | 39891.89 | 6.81 | Extracellular | -0.17 |
| [Soltu.DM.01G031240.1](http://spuddb.uga.edu/cgi-bin/annotation_report.cgi?orf=Soltu.DM.01G031240.1) | 1 | 315 | St2ODD20 | 35812.46 | 5.24 | Extracellular | -0.38 |
| [Soltu.DM.01G032750.1](http://spuddb.uga.edu/cgi-bin/annotation_report.cgi?orf=Soltu.DM.01G032750.1) | 1 | 365 | St2ODD22 | 42205.09 | 6.4 | Extracellular | -0.41 |
| [Soltu.DM.01G022530.1](http://spuddb.uga.edu/cgi-bin/annotation_report.cgi?orf=Soltu.DM.01G022530.1) | 1 | 363 | St2ODD16 | 41297.59 | 5.67 | Cytoplasmic | -0.25 |
| [Soltu.DM.01G008580.2](http://spuddb.uga.edu/cgi-bin/annotation_report.cgi?orf=Soltu.DM.01G008580.2) | 1 | 320 | St2ODD9 | 36444.5 | 5.38 | Extracellular | -0.27 |
| [Soltu.DM.01G033590.1](http://spuddb.uga.edu/cgi-bin/annotation_report.cgi?orf=Soltu.DM.01G033590.1) | 1 | 321 | St2ODD23 | 35855.83 | 5.29 | Extracellular | -0.21 |
| [Soltu.DM.01G008570.1](http://spuddb.uga.edu/cgi-bin/annotation_report.cgi?orf=Soltu.DM.01G008570.1) | 1 | 320 | St2ODD7 | 36444.5 | 5.38 | Extracellular | -0.27 |
| [Soltu.DM.01G030800.1](http://spuddb.uga.edu/cgi-bin/annotation_report.cgi?orf=Soltu.DM.01G030800.1) | 1 | 351 | St2ODD19 | 39578.39 | 6.16 | Extracellular | -0.23 |
| [Soltu.DM.01G002190.1](http://spuddb.uga.edu/cgi-bin/annotation_report.cgi?orf=Soltu.DM.01G002190.1) | 1 | 320 | St2ODD2 | 36380.46 | 5.45 | Extracellular | -0.25 |
| [Soltu.DM.01G002210.1](http://spuddb.uga.edu/cgi-bin/annotation_report.cgi?orf=Soltu.DM.01G002210.1) | 1 | 276 | St2ODD3 | 31501.83 | 5.38 | Extracellular | -0.39 |
| [Soltu.DM.01G031260.1](http://spuddb.uga.edu/cgi-bin/annotation_report.cgi?orf=Soltu.DM.01G031260.1) | 1 | 316 | St2ODD21 | 35907.4 | 5.15 | Extracellular | -0.36 |
| [Soltu.DM.01G014460.1](http://spuddb.uga.edu/cgi-bin/annotation_report.cgi?orf=Soltu.DM.01G014460.1) | 1 | 363 | St2ODD13 | 40859.68 | 5.75 | Cytoplasmic | -0.29 |
| [Soltu.DM.01G008560.1](http://spuddb.uga.edu/cgi-bin/annotation_report.cgi?orf=Soltu.DM.01G008560.1) | 1 | 320 | St2ODD5 | 36444.5 | 5.38 | Extracellular | -0.27 |
| [Soltu.DM.01G008560.2](http://spuddb.uga.edu/cgi-bin/annotation_report.cgi?orf=Soltu.DM.01G008560.2) | 1 | 275 | St2ODD6 | 31549.23 | 5.59 | Extracellular | -0.16 |
| [Soltu.DM.01G044880.1](http://spuddb.uga.edu/cgi-bin/annotation_report.cgi?orf=Soltu.DM.01G044880.1) | 1 | 353 | St2ODD24 | 40168.13 | 6.64 | Extracellular | -0.35 |
| [Soltu.DM.01G018850.1](http://spuddb.uga.edu/cgi-bin/annotation_report.cgi?orf=Soltu.DM.01G018850.1) | 1 | 349 | St2ODD15 | 39664.66 | 7.29 | Extracellular | -0.17 |
| [Soltu.DM.01G014230.1](http://spuddb.uga.edu/cgi-bin/annotation_report.cgi?orf=Soltu.DM.01G014230.1) | 1 | 354 | St2ODD12 | 40003.91 | 6.22 | Cytoplasmic | -0.23 |
| [Soltu.DM.01G002240.1](http://spuddb.uga.edu/cgi-bin/annotation_report.cgi?orf=Soltu.DM.01G002240.1) | 1 | 275 | St2ODD4 | 31305.42 | 5.01 | Extracellular | -0.37 |
| [Soltu.DM.01G044890.2](http://spuddb.uga.edu/cgi-bin/annotation_report.cgi?orf=Soltu.DM.01G044890.2) | 1 | 360 | St2ODD25 | 40880.52 | 6.21 | Cytoplasmic | -0.4 |
| [Soltu.DM.01G014000.1](http://spuddb.uga.edu/cgi-bin/annotation_report.cgi?orf=Soltu.DM.01G014000.1) | 1 | 314 | St2ODD11 | 35299.96 | 7.11 | Cytoplasmic | -0.11 |
| [Soltu.DM.01G048560.1](http://spuddb.uga.edu/cgi-bin/annotation_report.cgi?orf=Soltu.DM.01G048560.1) | 1 | 351 | St2ODD26 | 40601.26 | 6.21 | Extracellular | -0.57 |
| [Soltu.DM.01G008580.1](http://spuddb.uga.edu/cgi-bin/annotation_report.cgi?orf=Soltu.DM.01G008580.1) | 1 | 240 | St2ODD8 | 27459.24 | 4.97 | Extracellular | -0.28 |
| [Soltu.DM.02G023850.1](http://spuddb.uga.edu/cgi-bin/annotation_report.cgi?orf=Soltu.DM.02G023850.1) | 2 | 360 | St2ODD58 | 40572.3 | 5.41 | Extracellular | -0.46 |
| [Soltu.DM.02G014440.1](http://spuddb.uga.edu/cgi-bin/annotation_report.cgi?orf=Soltu.DM.02G014440.1) | 2 | 307 | St2ODD44 | 35059.23 | 4.98 | Cytoplasmic | -0.28 |
| [**Soltu.DM.02G013470.1**](http://spuddb.uga.edu/cgi-bin/annotation_report.cgi?orf=Soltu.DM.02G013470.1) | **2** | **340** | **St2ODD34** | 38069.58 | 5.94 | Extracellular | -0.18 |
| [Soltu.DM.02G014560.1](http://spuddb.uga.edu/cgi-bin/annotation_report.cgi?orf=Soltu.DM.02G014560.1) | 2 | 349 | St2ODD52 | 40129 | 5.33 | Cytoplasmic | -0.43 |
| [Soltu.DM.02G019740.1](http://spuddb.uga.edu/cgi-bin/annotation_report.cgi?orf=Soltu.DM.02G019740.1) | 2 | 359 | St2ODD55 | 41885.91 | 8.64 | Extracellular | -0.51 |
| [Soltu.DM.02G028350.2](http://spuddb.uga.edu/cgi-bin/annotation_report.cgi?orf=Soltu.DM.02G028350.2) | 2 | 330 | St2ODD61 | 36722.86 | 5.4 | Extracellular | -0.26 |
| [Soltu.DM.02G014420.5](http://spuddb.uga.edu/cgi-bin/annotation_report.cgi?orf=Soltu.DM.02G014420.5) | 2 | 524 | St2ODD38 | 59873.94 | 5.98 | Extracellular | -0.32 |
| [Soltu.DM.02G014420.6](http://spuddb.uga.edu/cgi-bin/annotation_report.cgi?orf=Soltu.DM.02G014420.6) | 2 | 568 | St2ODD39 | 4549.29 | 6.28 | Extracellular | -0.3 |
| [Soltu.DM.02G011120.1](http://spuddb.uga.edu/cgi-bin/annotation_report.cgi?orf=Soltu.DM.02G011120.1) | 2 | 301 | St2ODD32 | 33975.92 | 5.18 | Extracellular | -0.26 |
| [Soltu.DM.02G014420.1](http://spuddb.uga.edu/cgi-bin/annotation_report.cgi?orf=Soltu.DM.02G014420.1) | 2 | 359 | St2ODD40 | 41020.02 | 5.77 | Cytoplasmic | -0.41 |
| [Soltu.DM.02G028330.1](http://spuddb.uga.edu/cgi-bin/annotation_report.cgi?orf=Soltu.DM.02G028330.1) | 2 | 373 | St2ODD60 | 42000.92 | 5.32 | Extracellular | -0.15 |
| [Soltu.DM.02G014410.1](http://spuddb.uga.edu/cgi-bin/annotation_report.cgi?orf=Soltu.DM.02G014410.1) | 2 | 366 | St2ODD35 | 32196.62 | 5.47 | Extracellular | -0.25 |
| [Soltu.DM.02G020690.2](http://spuddb.uga.edu/cgi-bin/annotation_report.cgi?orf=Soltu.DM.02G020690.2) | 2 | 232 | St2ODD57 | 26332.37 | 5.07 | Extracellular | -0.24 |
| [Soltu.DM.02G014430.2](http://spuddb.uga.edu/cgi-bin/annotation_report.cgi?orf=Soltu.DM.02G014430.2) | 2 | 297 | St2ODD43 | 34126.74 | 5.14 | Extracellular | -0.14 |
| [Soltu.DM.02G014470.1](http://spuddb.uga.edu/cgi-bin/annotation_report.cgi?orf=Soltu.DM.02G014470.1) | 2 | 353 | St2ODD46 | 39998 | 5.1 | Cytoplasmic | -0.24 |
| [Soltu.DM.02G014570.1](http://spuddb.uga.edu/cgi-bin/annotation_report.cgi?orf=Soltu.DM.02G014570.1) | 2 | 621 | St2ODD53 | 71122.1 | 5.3 | Extracellular | -0.3 |
| [**Soltu.DM.02G014420.3**](http://spuddb.uga.edu/cgi-bin/annotation_report.cgi?orf=Soltu.DM.02G014420.3) | **2** | **362** | **St2ODD41** | 41323.39 | 5.77 | Cytoplasmic | -0.39 |
| [Soltu.DM.02G005160.1](http://spuddb.uga.edu/cgi-bin/annotation_report.cgi?orf=Soltu.DM.02G005160.1) | 2 | 198 | St2ODD30 | 22393.82 | 6.04 | Extracellular | -0.03 |
| [Soltu.DM.02G023850.2](http://spuddb.uga.edu/cgi-bin/annotation_report.cgi?orf=Soltu.DM.02G023850.2) | 2 | 273 | St2ODD59 | 30858.15 | 5.03 | Extracellular | -0.36 |
| [Soltu.DM.02G005130.1](http://spuddb.uga.edu/cgi-bin/annotation_report.cgi?orf=Soltu.DM.02G005130.1) | 2 | 350 | St2ODD29 | 39696.51 | 6.34 | Extracellular | -0.18 |
| [Soltu.DM.02G011100.1](http://spuddb.uga.edu/cgi-bin/annotation_report.cgi?orf=Soltu.DM.02G011100.1) | 2 | 301 | St2ODD31 | 33978.01 | 5.28 | Extracellular | -0.25 |
| [Soltu.DM.02G014420.4](http://spuddb.uga.edu/cgi-bin/annotation_report.cgi?orf=Soltu.DM.02G014420.4) | 2 | 565 | St2ODD37 | 64245.92 | 6.28 | Extracellular | -0.31 |
| [Soltu.DM.02G014540.1](http://spuddb.uga.edu/cgi-bin/annotation_report.cgi?orf=Soltu.DM.02G014540.1) | 2 | 360 | St2ODD50 | 41191.13 | 5.09 | Extracellular | -0.34 |
| [Soltu.DM.02G014520.1](http://spuddb.uga.edu/cgi-bin/annotation_report.cgi?orf=Soltu.DM.02G014520.1) | 2 | 546 | St2ODD48 | 62096.28 | 8.15 | Extracellular | -0.42 |
| [Soltu.DM.02G014550.1](http://spuddb.uga.edu/cgi-bin/annotation_report.cgi?orf=Soltu.DM.02G014550.1) | 2 | 955 | St2ODD51 | 108596.08 | 5.75 | Extracellular | -0.26 |
| [Soltu.DM.02G014430.1](http://spuddb.uga.edu/cgi-bin/annotation_report.cgi?orf=Soltu.DM.02G014430.1) | 2 | 355 | St2ODD42 | 40462.72 | 5.7 | Extracellular | -0.35 |
| [Soltu.DM.02G014530.1](http://spuddb.uga.edu/cgi-bin/annotation_report.cgi?orf=Soltu.DM.02G014530.1) | 2 | 361 | St2ODD49 | 40910.05 | 5.52 | Extracellular | -0.25 |
| [Soltu.DM.02G028350.3](http://spuddb.uga.edu/cgi-bin/annotation_report.cgi?orf=Soltu.DM.02G028350.3) | 2 | 330 | St2ODD62 | 36722.86 | 5.4 | Extracellular | -0.26 |
| [Soltu.DM.02G014450.1](http://spuddb.uga.edu/cgi-bin/annotation_report.cgi?orf=Soltu.DM.02G014450.1) | 2 | 358 | St2ODD45 | 41006.05 | 5.86 | Extracellular | -0.41 |
| [Soltu.DM.02G014580.2](http://spuddb.uga.edu/cgi-bin/annotation_report.cgi?orf=Soltu.DM.02G014580.2) | 2 | 910 | St2ODD54 | 103324.72 | 7.21 | Extracellular | -0.52 |
| [Soltu.DM.02G013120.1](http://spuddb.uga.edu/cgi-bin/annotation_report.cgi?orf=Soltu.DM.02G013120.1) | 2 | 341 | St2ODD33 | 38912.56 | 6.23 | Cytoplasmic | -0.3 |
| [Soltu.DM.02G004480.1](http://spuddb.uga.edu/cgi-bin/annotation_report.cgi?orf=Soltu.DM.02G004480.1) | 2 | 319 | St2ODD28 | 36386.96 | 5.21 | Extracellular | -0.34 |
| [Soltu.DM.02G014420.2](http://spuddb.uga.edu/cgi-bin/annotation_report.cgi?orf=Soltu.DM.02G014420.2) | 2 | 357 | St2ODD36 | 40854.9 | 6.29 | Extracellular | -0.41 |
| [Soltu.DM.02G020690.1](http://spuddb.uga.edu/cgi-bin/annotation_report.cgi?orf=Soltu.DM.02G020690.1) | 2 | 320 | St2ODD56 | 36269.64 | 5.13 | Cytoplasmic | -0.35 |
| [Soltu.DM.02G014490.1](http://spuddb.uga.edu/cgi-bin/annotation_report.cgi?orf=Soltu.DM.02G014490.1) | 2 | 366 | St2ODD47 | 41318.59 | 5.1 | Cytoplasmic | -0.27 |
| [Soltu.DM.03G021450.3](http://spuddb.uga.edu/cgi-bin/annotation_report.cgi?orf=Soltu.DM.03G021450.3) | 3 | 337 | St2ODD72 | 38223.56 | 5.62 | Cytoplasmic | -0.35 |
| [Soltu.DM.03G016400.2](http://spuddb.uga.edu/cgi-bin/annotation_report.cgi?orf=Soltu.DM.03G016400.2) | 3 | 296 | St2ODD68 | 33718.26 | 5.42 | Extracellular | -0.26 |
| [Soltu.DM.03G035700.1](http://spuddb.uga.edu/cgi-bin/annotation_report.cgi?orf=Soltu.DM.03G035700.1) | 3 | 382 | St2ODD73 | 43322.43 | 5.54 | Extracellular | -0.33 |
| [Soltu.DM.03G021450.2](http://spuddb.uga.edu/cgi-bin/annotation_report.cgi?orf=Soltu.DM.03G021450.2) | 3 | 337 | St2ODD70 | 38223.56 | 5.62 | Cytoplasmic | -0.35 |
| [Soltu.DM.03G013090.3](http://spuddb.uga.edu/cgi-bin/annotation_report.cgi?orf=Soltu.DM.03G013090.3) | 3 | 273 | St2ODD65 | 30983.21 | 6.5 | Cytoplasmic | -0.44 |
| [Soltu.DM.03G013240.1](http://spuddb.uga.edu/cgi-bin/annotation_report.cgi?orf=Soltu.DM.03G013240.1) | 3 | 362 | St2ODD66 | 40845.6 | 5.36 | Cytoplasmic | -0.32 |
| [Soltu.DM.03G021450.1](http://spuddb.uga.edu/cgi-bin/annotation_report.cgi?orf=Soltu.DM.03G021450.1) | 3 | 337 | St2ODD71 | 38223.56 | 5.62 | Cytoplasmic | -0.35 |
| [Soltu.DM.03G013090.1](http://spuddb.uga.edu/cgi-bin/annotation_report.cgi?orf=Soltu.DM.03G013090.1) | 3 | 356 | St2ODD63 | 40583.27 | 7.73 | Cytoplasmic | -0.5 |
| [Soltu.DM.03G017930.1](http://spuddb.uga.edu/cgi-bin/annotation_report.cgi?orf=Soltu.DM.03G017930.1) | 3 | 318 | St2ODD69 | 35925.15 | 6.52 | Extracellular | -0.21 |
| [Soltu.DM.03G016400.1](http://spuddb.uga.edu/cgi-bin/annotation_report.cgi?orf=Soltu.DM.03G016400.1) | 3 | 378 | St2ODD67 | 43143.76 | 5.8 | Extracellular | -0.4 |
| [Soltu.DM.03G013090.2](http://spuddb.uga.edu/cgi-bin/annotation_report.cgi?orf=Soltu.DM.03G013090.2) | 3 | 273 | St2ODD64 | 31041.29 | 6.75 | Cytoplasmic | -0.46 |
| [Soltu.DM.04G004570.2](http://spuddb.uga.edu/cgi-bin/annotation_report.cgi?orf=Soltu.DM.04G004570.2) | 4 | 266 | St2ODD75 | 30358.79 | 5.42 | Cytoplasmic | -0.26 |
| [**Soltu.DM.04G004570.1**](http://spuddb.uga.edu/cgi-bin/annotation_report.cgi?orf=Soltu.DM.04G004570.1) | **4** | **333** | **St2ODD76** | 38187.8 | 6.33 | Cytoplasmic | -0.32 |
| [Soltu.DM.04G006650.1](http://spuddb.uga.edu/cgi-bin/annotation_report.cgi?orf=Soltu.DM.04G006650.1) | 4 | 286 | St2ODD77 | 32196.62 | 5.47 | Cytoplasmic | -0.37 |
| [Soltu.DM.04G006660.1](http://spuddb.uga.edu/cgi-bin/annotation_report.cgi?orf=Soltu.DM.04G006660.1) | 4 | 364 | St2ODD80 | 41179.06 | 5.5 | Cytoplasmic | -0.25 |
| [Soltu.DM.04G006650.2](http://spuddb.uga.edu/cgi-bin/annotation_report.cgi?orf=Soltu.DM.04G006650.2) | 4 | 294 | St2ODD78 | 33121.63 | 5.37 | Cytoplasmic | -0.33 |
| [Soltu.DM.04G006650.3](http://spuddb.uga.edu/cgi-bin/annotation_report.cgi?orf=Soltu.DM.04G006650.3) | 4 | 368 | St2ODD79 | 41647.36 | 5.87 | Cytoplasmic | -0.37 |
| [Soltu.DM.04G003360.1](http://spuddb.uga.edu/cgi-bin/annotation_report.cgi?orf=Soltu.DM.04G003360.1) | 4 | 366 | St2ODD74 | 41334.01 | 5.53 | Cytoplasmic | -0.4 |
| [Soltu.DM.05G023320.1](http://spuddb.uga.edu/cgi-bin/annotation_report.cgi?orf=Soltu.DM.05G023320.1) | 5 | 349 | St2ODD83 | 39308.81 | 7.19 | Extracellular | -0.31 |
| [Soltu.DM.05G013110.1](http://spuddb.uga.edu/cgi-bin/annotation_report.cgi?orf=Soltu.DM.05G013110.1) | 5 | 324 | St2ODD81 | 36496.32 | 5.09 | Extracellular | -0.34 |
| [Soltu.DM.05G022760.1](http://spuddb.uga.edu/cgi-bin/annotation_report.cgi?orf=Soltu.DM.05G022760.1) | 5 | 349 | St2ODD82 | 39713.64 | 6.49 | Extracellular | -0.14 |
| [Soltu.DM.06G034290.1](http://spuddb.uga.edu/cgi-bin/annotation_report.cgi?orf=Soltu.DM.06G034290.1) | 6 | 337 | St2ODD114 | 38441.89 | 6.1 | Cytoplasmic | -0.31 |
| [**Soltu.DM.06G023440.1**](http://spuddb.uga.edu/cgi-bin/annotation_report.cgi?orf=Soltu.DM.06G023440.1) | **6** | **372** | **St2ODD99** | 41315.96 | 8.14 | Extracellular | -0.33 |
| [Soltu.DM.06G016970.1](http://spuddb.uga.edu/cgi-bin/annotation_report.cgi?orf=Soltu.DM.06G016970.1) | 6 | 313 | St2ODD93 | 35497.3 | 5.53 | Cytoplasmic | -0.45 |
| [Soltu.DM.06G018100.2](http://spuddb.uga.edu/cgi-bin/annotation_report.cgi?orf=Soltu.DM.06G018100.2) | 6 | 283 | St2ODD95 | 32293.1 | 6.8 | Extracellular | -0.27 |
| [Soltu.DM.06G005250.1](http://spuddb.uga.edu/cgi-bin/annotation_report.cgi?orf=Soltu.DM.06G005250.1) | 6 | 209 | St2ODD88 | 23981.44 | 5.88 | Cytoplasmic | -0.39 |
| [Soltu.DM.06G023930.1](http://spuddb.uga.edu/cgi-bin/annotation_report.cgi?orf=Soltu.DM.06G023930.1) | 6 | 331 | St2ODD108 | 38139.42 | 5.04 | Extracellular | -0.5 |
| [Soltu.DM.06G023450.1](http://spuddb.uga.edu/cgi-bin/annotation_report.cgi?orf=Soltu.DM.06G023450.1) | 6 | 313 | St2ODD100 | 35874.83 | 5.71 | Extracellular | -0.43 |
| [Soltu.DM.06G005240.1](http://spuddb.uga.edu/cgi-bin/annotation_report.cgi?orf=Soltu.DM.06G005240.1) | 6 | 357 | St2ODD87 | 40770.61 | 5.45 | Extracellular | -0.37 |
| [Soltu.DM.06G023470.1](http://spuddb.uga.edu/cgi-bin/annotation_report.cgi?orf=Soltu.DM.06G023470.1) | 6 | 314 | St2ODD101 | 36336.82 | 6.83 | Extracellular | -0.42 |
| [**Soltu.DM.06G014640.1**](http://spuddb.uga.edu/cgi-bin/annotation_report.cgi?orf=Soltu.DM.06G014640.1) | **6** | **361** | **St2ODD91** | 40309.58 | 5.72 | Cytoplasmic | -0.31 |
| [Soltu.DM.06G023520.1](http://spuddb.uga.edu/cgi-bin/annotation_report.cgi?orf=Soltu.DM.06G023520.1) | 6 | 253 | St2ODD104 | 29231.27 | 5.94 | Extracellular | -0.44 |
| [Soltu.DM.06G005230.1](http://spuddb.uga.edu/cgi-bin/annotation_report.cgi?orf=Soltu.DM.06G005230.1) | 6 | 327 | St2ODD86 | 37518.04 | 6.09 | Cytoplasmic | -0.35 |
| [Soltu.DM.06G023290.1](http://spuddb.uga.edu/cgi-bin/annotation_report.cgi?orf=Soltu.DM.06G023290.1) | 6 | 323 | St2ODD98 | 37061.02 | 5.89 | Extracellular | -0.35 |
| [Soltu.DM.06G005200.1](http://spuddb.uga.edu/cgi-bin/annotation_report.cgi?orf=Soltu.DM.06G005200.1) | 6 | 357 | St2ODD85 | 40498.35 | 5.65 | Extracellular | -0.35 |
| [Soltu.DM.06G012790.2](http://spuddb.uga.edu/cgi-bin/annotation_report.cgi?orf=Soltu.DM.06G012790.2) | 6 | 303 | St2ODD90 | 34429.39 | 6.35 | Extracellular | -0.28 |
| [Soltu.DM.06G023560.1](http://spuddb.uga.edu/cgi-bin/annotation_report.cgi?orf=Soltu.DM.06G023560.1) | 6 | 318 | St2ODD106 | 36754.97 | 5.53 | Extracellular | -0.31 |
| [Soltu.DM.06G023470.2](http://spuddb.uga.edu/cgi-bin/annotation_report.cgi?orf=Soltu.DM.06G023470.2) | 6 | 255 | St2ODD102 | 29374.78 | 6.66 | Extracellular | -0.35 |
| [Soltu.DM.06G023940.1](http://spuddb.uga.edu/cgi-bin/annotation_report.cgi?orf=Soltu.DM.06G023940.1) | 6 | 376 | St2ODD109 | 42885.54 | 4.75 | Extracellular | -0.46 |
| [Soltu.DM.06G018740.1](http://spuddb.uga.edu/cgi-bin/annotation_report.cgi?orf=Soltu.DM.06G018740.1) | 6 | 314 | St2ODD97 | 36206.62 | 6.5 | Extracellular | -0.37 |
| [Soltu.DM.06G018100.1](http://spuddb.uga.edu/cgi-bin/annotation_report.cgi?orf=Soltu.DM.06G018100.1) | 6 | 311 | St2ODD94 | 35410.53 | 6.16 | Extracellular | -0.29 |
| [Soltu.DM.06G032330.1](http://spuddb.uga.edu/cgi-bin/annotation_report.cgi?orf=Soltu.DM.06G032330.1) | 6 | 355 | St2ODD113 | 40772.23 | 5.95 | Extracellular | -0.39 |
| [Soltu.DM.06G014640.2](http://spuddb.uga.edu/cgi-bin/annotation_report.cgi?orf=Soltu.DM.06G014640.2) | 6 | 312 | St2ODD92 | 35476.41 | 5.24 | Cytoplasmic | -0.22 |
| [Soltu.DM.06G026020.1](http://spuddb.uga.edu/cgi-bin/annotation_report.cgi?orf=Soltu.DM.06G026020.1) | 6 | 213 | St2ODD110 | 24195.68 | 4.91 | Extracellular | -0.31 |
| [Soltu.DM.06G018100.3](http://spuddb.uga.edu/cgi-bin/annotation_report.cgi?orf=Soltu.DM.06G018100.3) | 6 | 269 | St2ODD96 | 30722.21 | 6.51 | Extracellular | -0.3 |
| [Soltu.DM.06G028900.1](http://spuddb.uga.edu/cgi-bin/annotation_report.cgi?orf=Soltu.DM.06G028900.1) | 6 | 394 | St2ODD112 | 44858.21 | 5.45 | Extracellular | -0.45 |
| [Soltu.DM.06G023510.1](http://spuddb.uga.edu/cgi-bin/annotation_report.cgi?orf=Soltu.DM.06G023510.1) | 6 | 315 | St2ODD103 | 36166.23 | 5.83 | Extracellular | -0.38 |
| [Soltu.DM.06G012790.1](http://spuddb.uga.edu/cgi-bin/annotation_report.cgi?orf=Soltu.DM.06G012790.1) | 6 | 377 | St2ODD89 | 42971.08 | 6.89 | Extracellular | -0.34 |
| [Soltu.DM.06G023570.1](http://spuddb.uga.edu/cgi-bin/annotation_report.cgi?orf=Soltu.DM.06G023570.1) | 6 | 317 | St2ODD107 | 36020.93 | 5.27 | Extracellular | -0.31 |
| [Soltu.DM.06G004570.1](http://spuddb.uga.edu/cgi-bin/annotation_report.cgi?orf=Soltu.DM.06G004570.1) | 6 | 357 | St2ODD84 | 40388.18 | 5.77 | Cytoplasmic | -0.4 |
| [Soltu.DM.06G023530.1](http://spuddb.uga.edu/cgi-bin/annotation_report.cgi?orf=Soltu.DM.06G023530.1) | 6 | 314 | St2ODD105 | 36322.62 | 6.21 | Extracellular | -0.38 |
| [Soltu.DM.06G028410.1](http://spuddb.uga.edu/cgi-bin/annotation_report.cgi?orf=Soltu.DM.06G028410.1) | 6 | 342 | St2ODD111 | 39115.47 | 5.28 | Cytoplasmic | -0.4 |
| [Soltu.DM.07G022710.1](http://spuddb.uga.edu/cgi-bin/annotation_report.cgi?orf=Soltu.DM.07G022710.1) | 7 | 330 | St2ODD125 | 37118.67 | 8.23 | Extracellular | -0.22 |
| [**Soltu.DM.07G011040.1**](http://spuddb.uga.edu/cgi-bin/annotation_report.cgi?orf=Soltu.DM.07G011040.1) | **7** | **301** | **St2ODD115** | 34314.19 | 5.77 | Cytoplasmic | -0.41 |
| [Soltu.DM.07G016750.1](http://spuddb.uga.edu/cgi-bin/annotation_report.cgi?orf=Soltu.DM.07G016750.1) | 7 | 315 | St2ODD118 | 35744.92 | 5.18 | Cytoplasmic | -0.4 |
| [Soltu.DM.07G018960.1](http://spuddb.uga.edu/cgi-bin/annotation_report.cgi?orf=Soltu.DM.07G018960.1) | 7 | 399 | St2ODD122 | 44517.24 | 6.87 | Cytoplasmic | -0.19 |
| [Soltu.DM.07G022720.2](http://spuddb.uga.edu/cgi-bin/annotation_report.cgi?orf=Soltu.DM.07G022720.2) | 7 | 276 | St2ODD128 | 30819.03 | 4.9 | Extracellular | -0.12 |
| [**Soltu.DM.07G014170.1**](http://spuddb.uga.edu/cgi-bin/annotation_report.cgi?orf=Soltu.DM.07G014170.1) | **7** | **341** | **St2ODD116** | 37881 | 5.54 | Cytoplasmic | -0.26 |
| [Soltu.DM.07G018990.1](http://spuddb.uga.edu/cgi-bin/annotation_report.cgi?orf=Soltu.DM.07G018990.1) | 7 | 360 | St2ODD123 | 40355.22 | 6.87 | Cytoplasmic | -0.37 |
| [Soltu.DM.07G022710.2](http://spuddb.uga.edu/cgi-bin/annotation_report.cgi?orf=Soltu.DM.07G022710.2) | 7 | 273 | St2ODD126 | 30400.81 | 6.09 | Extracellular | -0.14 |
| [Soltu.DM.07G022700.1](http://spuddb.uga.edu/cgi-bin/annotation_report.cgi?orf=Soltu.DM.07G022700.1) | 7 | 324 | St2ODD124 | 36243.27 | 5.67 | Extracellular | -0.2 |
| [Soltu.DM.07G018950.1](http://spuddb.uga.edu/cgi-bin/annotation_report.cgi?orf=Soltu.DM.07G018950.1) | 7 | 502 | St2ODD121 | 56341.73 | 8.94 | Extracellular | -0.33 |
| [Soltu.DM.07G016750.2](http://spuddb.uga.edu/cgi-bin/annotation_report.cgi?orf=Soltu.DM.07G016750.2) | 7 | 236 | St2ODD119 | 26791.68 | 4.92 | Cytoplasmic | -0.33 |
| [Soltu.DM.07G016780.1](http://spuddb.uga.edu/cgi-bin/annotation_report.cgi?orf=Soltu.DM.07G016780.1) | 7 | 318 | St2ODD120 | 36156.33 | 5 | Cytoplasmic | -0.35 |
| [Soltu.DM.07G014170.2](http://spuddb.uga.edu/cgi-bin/annotation_report.cgi?orf=Soltu.DM.07G014170.2) | 7 | 288 | St2ODD117 | 31912.35 | 5.04 | Extracellular | -0.11 |
| [Soltu.DM.07G022720.1](http://spuddb.uga.edu/cgi-bin/annotation_report.cgi?orf=Soltu.DM.07G022720.1) | 7 | 334 | St2ODD127 | 37567.94 | 5.78 | Extracellular | -0.22 |
| [Soltu.DM.08G006960.1](http://spuddb.uga.edu/cgi-bin/annotation_report.cgi?orf=Soltu.DM.08G006960.1) | 8 | 357 | St2ODD131 | 40025.03 | 7.6 | Extracellular | -0.22 |
| [Soltu.DM.08G024340.1](http://spuddb.uga.edu/cgi-bin/annotation_report.cgi?orf=Soltu.DM.08G024340.1) | 8 | 296 | St2ODD133 | 34099.23 | 5.31 | Extracellular | -0.32 |
| [Soltu.DM.08G024070.1](http://spuddb.uga.edu/cgi-bin/annotation_report.cgi?orf=Soltu.DM.08G024070.1) | 8 | 248 | St2ODD132 | 28171.68 | 8.44 | Extracellular | -0.14 |
| [Soltu.DM.08G001760.1](http://spuddb.uga.edu/cgi-bin/annotation_report.cgi?orf=Soltu.DM.08G001760.1) | 8 | 353 | St2ODD130 | 40090.58 | 6.53 | Cytoplasmic | -0.42 |
| [Soltu.DM.08G001750.1](http://spuddb.uga.edu/cgi-bin/annotation_report.cgi?orf=Soltu.DM.08G001750.1) | 8 | 253 | St2ODD129 | 28761.75 | 5.74 | Cytoplasmic | -0.35 |
| [Soltu.DM.08G026700.1](http://spuddb.uga.edu/cgi-bin/annotation_report.cgi?orf=Soltu.DM.08G026700.1) | 8 | 455 | St2ODD134 | 51331.63 | 5.27 | Cytoplasmic | -0.48 |
| [Soltu.DM.09G026410.2](http://spuddb.uga.edu/cgi-bin/annotation_report.cgi?orf=Soltu.DM.09G026410.2) | 9 | 306 | St2ODD167 | 34566.38 | 5.11 | Cytoplasmic | -0.34 |
| [Soltu.DM.09G021150.1](http://spuddb.uga.edu/cgi-bin/annotation_report.cgi?orf=Soltu.DM.09G021150.1) | 9 | 329 | St2ODD147 | 36963.28 | 5.64 | Cytoplasmic | -0.22 |
| [Soltu.DM.09G026290.2](http://spuddb.uga.edu/cgi-bin/annotation_report.cgi?orf=Soltu.DM.09G026290.2) | 9 | 368 | St2ODD159 | 41552.49 | 5.6 | Cytoplasmic | -0.22 |
| [Soltu.DM.09G026390.3](http://spuddb.uga.edu/cgi-bin/annotation_report.cgi?orf=Soltu.DM.09G026390.3) | 9 | 298 | St2ODD165 | 33543.43 | 5.4 | Cytoplasmic | -0.29 |
| [Soltu.DM.09G002960.4](http://spuddb.uga.edu/cgi-bin/annotation_report.cgi?orf=Soltu.DM.09G002960.4) | 9 | 370 | St2ODD139 | 41754.54 | 5.43 | Cytoplasmic | -0.32 |
| [**Soltu.DM.09G026220.3**](http://spuddb.uga.edu/cgi-bin/annotation_report.cgi?orf=Soltu.DM.09G026220.3) | **9** | **310** | **St2ODD153** | 34800.56 | 4.87 | Cytoplasmic | -0.24 |
| [Soltu.DM.09G021160.1](http://spuddb.uga.edu/cgi-bin/annotation_report.cgi?orf=Soltu.DM.09G021160.1) | 9 | 329 | St2ODD148 | 37014.37 | 5.46 | Cytoplasmic | -0.25 |
| [Soltu.DM.09G018070.1](http://spuddb.uga.edu/cgi-bin/annotation_report.cgi?orf=Soltu.DM.09G018070.1) | 9 | 363 | St2ODD146 | 41178.54 | 5.85 | Extracellular | -0.37 |
| [Soltu.DM.09G002950.1](http://spuddb.uga.edu/cgi-bin/annotation_report.cgi?orf=Soltu.DM.09G002950.1) | 9 | 300 | St2ODD135 | 33617.78 | 8.6 | Cytoplasmic | -0.29 |
| [Soltu.DM.09G021170.1](http://spuddb.uga.edu/cgi-bin/annotation_report.cgi?orf=Soltu.DM.09G021170.1) | 9 | 399 | St2ODD149 | 44971.98 | 4.82 | Extracellular | -0.3 |
| [Soltu.DM.09G003020.1](http://spuddb.uga.edu/cgi-bin/annotation_report.cgi?orf=Soltu.DM.09G003020.1) | 9 | 359 | St2ODD142 | 40735.67 | 6.14 | Cytoplasmic | -0.31 |
| [Soltu.DM.09G026220.2](http://spuddb.uga.edu/cgi-bin/annotation_report.cgi?orf=Soltu.DM.09G026220.2) | 9 | 295 | St2ODD152 | 32940.46 | 4.75 | Cytoplasmic | -0.26 |
| [Soltu.DM.09G026230.1](http://spuddb.uga.edu/cgi-bin/annotation_report.cgi?orf=Soltu.DM.09G026230.1) | 9 | 295 | St2ODD155 | 32946.46 | 4.71 | Cytoplasmic | -0.24 |
| [Soltu.DM.09G002970.1](http://spuddb.uga.edu/cgi-bin/annotation_report.cgi?orf=Soltu.DM.09G002970.1) | 9 | 370 | St2ODD140 | 41986.65 | 5.97 | Cytoplasmic | -0.4 |
| [Soltu.DM.09G026280.1](http://spuddb.uga.edu/cgi-bin/annotation_report.cgi?orf=Soltu.DM.09G026280.1) | 9 | 352 | St2ODD158 | 39327.1 | 5.47 | Cytoplasmic | -0.25 |
| [Soltu.DM.09G029390.1](http://spuddb.uga.edu/cgi-bin/annotation_report.cgi?orf=Soltu.DM.09G029390.1) | 9 | 355 | St2ODD169 | 40404.34 | 5.68 | Cytoplasmic | -0.3 |
| [Soltu.DM.09G026370.1](http://spuddb.uga.edu/cgi-bin/annotation_report.cgi?orf=Soltu.DM.09G026370.1) | 9 | 335 | St2ODD162 | 37897.66 | 6.02 | Cytoplasmic | -0.28 |
| [Soltu.DM.09G026200.1](http://spuddb.uga.edu/cgi-bin/annotation_report.cgi?orf=Soltu.DM.09G026200.1) | 9 | 382 | St2ODD150 | 43343.4 | 5.54 | Cytoplasmic | -0.35 |
| [**Soltu.DM.09G026220.1**](http://spuddb.uga.edu/cgi-bin/annotation_report.cgi?orf=Soltu.DM.09G026220.1) | **9** | **365** | **St2ODD151** | 40594.25 | 5.1 | Cytoplasmic | -0.24 |
| [Soltu.DM.09G002960.1](http://spuddb.uga.edu/cgi-bin/annotation_report.cgi?orf=Soltu.DM.09G002960.1) | 9 | 292 | St2ODD136 | 32746.16 | 4.88 | Cytoplasmic | -0.3 |
| [Soltu.DM.09G026230.2](http://spuddb.uga.edu/cgi-bin/annotation_report.cgi?orf=Soltu.DM.09G026230.2) | 9 | 373 | St2ODD156 | 41855.65 | 5.12 | Cytoplasmic | -0.29 |
| [Soltu.DM.09G026360.1](http://spuddb.uga.edu/cgi-bin/annotation_report.cgi?orf=Soltu.DM.09G026360.1) | 9 | 356 | St2ODD161 | 39675.47 | 5.28 | Cytoplasmic | -0.18 |
| [Soltu.DM.09G026250.1](http://spuddb.uga.edu/cgi-bin/annotation_report.cgi?orf=Soltu.DM.09G026250.1) | 9 | 345 | St2ODD157 | 38890.09 | 5.97 | Cytoplasmic | -0.39 |
| [Soltu.DM.09G016350.1](http://spuddb.uga.edu/cgi-bin/annotation_report.cgi?orf=Soltu.DM.09G016350.1) | 9 | 338 | St2ODD145 | 38189.22 | 5.64 | Extracellular | -0.27 |
| [Soltu.DM.09G002960.3](http://spuddb.uga.edu/cgi-bin/annotation_report.cgi?orf=Soltu.DM.09G002960.3) | 9 | 370 | St2ODD138 | 41754.54 | 5.43 | Cytoplasmic | -0.32 |
| [Soltu.DM.09G026290.1](http://spuddb.uga.edu/cgi-bin/annotation_report.cgi?orf=Soltu.DM.09G026290.1) | 9 | 368 | St2ODD160 | 41552.49 | 5.6 | Cytoplasmic | -0.22 |
| [Soltu.DM.09G026220.4](http://spuddb.uga.edu/cgi-bin/annotation_report.cgi?orf=Soltu.DM.09G026220.4) | 9 | 373 | St2ODD154 | 41447.24 | 5.04 | Cytoplasmic | -0.22 |
| [Soltu.DM.09G003000.1](http://spuddb.uga.edu/cgi-bin/annotation_report.cgi?orf=Soltu.DM.09G003000.1) | 9 | 359 | St2ODD141 | 40869.9 | 6.46 | Cytoplasmic | -0.3 |
| [Soltu.DM.09G004900.1](http://spuddb.uga.edu/cgi-bin/annotation_report.cgi?orf=Soltu.DM.09G004900.1) | 9 | 382 | St2ODD143 | 43303.44 | 5.66 | Cytoplasmic | -0.34 |
| [Soltu.DM.09G002960.2](http://spuddb.uga.edu/cgi-bin/annotation_report.cgi?orf=Soltu.DM.09G002960.2) | 9 | 306 | St2ODD137 | 34369.96 | 4.95 | Cytoplasmic | -0.27 |
| [Soltu.DM.09G026410.3](http://spuddb.uga.edu/cgi-bin/annotation_report.cgi?orf=Soltu.DM.09G026410.3) | 9 | 358 | St2ODD168 | 40278.34 | 5.45 | Cytoplasmic | -0.26 |
| [Soltu.DM.09G026390.2](http://spuddb.uga.edu/cgi-bin/annotation_report.cgi?orf=Soltu.DM.09G026390.2) | 9 | 359 | St2ODD164 | 40242.22 | 5.38 | Cytoplasmic | -0.23 |
| [Soltu.DM.09G010160.1](http://spuddb.uga.edu/cgi-bin/annotation_report.cgi?orf=Soltu.DM.09G010160.1) | 9 | 360 | St2ODD144 | 41322.91 | 6.23 | Extracellular | -0.39 |
| [Soltu.DM.09G026410.1](http://spuddb.uga.edu/cgi-bin/annotation_report.cgi?orf=Soltu.DM.09G026410.1) | 9 | 358 | St2ODD166 | 40278.34 | 5.45 | Cytoplasmic | -0.26 |
| [Soltu.DM.09G026380.1](http://spuddb.uga.edu/cgi-bin/annotation_report.cgi?orf=Soltu.DM.09G026380.1) | 9 | 358 | St2ODD163 | 40527.37 | 5.24 | Cytoplasmic | -0.31 |
| [Soltu.DM.10G019640.1](http://spuddb.uga.edu/cgi-bin/annotation_report.cgi?orf=Soltu.DM.10G019640.1) | 10 | 366 | St2ODD176 | 41400.16 | 6.28 | Cytoplasmic | -0.4 |
| [Soltu.DM.10G022640.1](http://spuddb.uga.edu/cgi-bin/annotation_report.cgi?orf=Soltu.DM.10G022640.1) | 10 | 360 | St2ODD182 | 40905.66 | 8.37 | Cytoplasmic | -0.39 |
| [Soltu.DM.10G019920.2](http://spuddb.uga.edu/cgi-bin/annotation_report.cgi?orf=Soltu.DM.10G019920.2) | 10 | 310 | St2ODD181 | 35534.7 | 4.93 | Extracellular | -0.18 |
| [Soltu.DM.10G019650.1](http://spuddb.uga.edu/cgi-bin/annotation_report.cgi?orf=Soltu.DM.10G019650.1) | 10 | 361 | St2ODD178 | 41041.03 | 6.18 | Cytoplasmic | -0.36 |
| [Soltu.DM.10G019910.1](http://spuddb.uga.edu/cgi-bin/annotation_report.cgi?orf=Soltu.DM.10G019910.1) | 10 | 348 | St2ODD179 | 39804.62 | 5.46 | Cytoplasmic | -0.33 |
| [Soltu.DM.10G019920.1](http://spuddb.uga.edu/cgi-bin/annotation_report.cgi?orf=Soltu.DM.10G019920.1) | 10 | 347 | St2ODD180 | 39520.19 | 4.96 | Extracellular | -0.28 |
| [Soltu.DM.10G007320.1](http://spuddb.uga.edu/cgi-bin/annotation_report.cgi?orf=Soltu.DM.10G007320.1) | 10 | 327 | St2ODD173 | 36889.44 | 6.5 | Extracellular | -0.23 |
| [Soltu.DM.10G000140.1](http://spuddb.uga.edu/cgi-bin/annotation_report.cgi?orf=Soltu.DM.10G000140.1) | 10 | 338 | St2ODD170 | 37911.74 | 4.68 | Extracellular | -0.29 |
| [Soltu.DM.10G027020.1](http://spuddb.uga.edu/cgi-bin/annotation_report.cgi?orf=Soltu.DM.10G027020.1) | 10 | 313 | St2ODD184 | 35464.75 | 5.34 | Extracellular | -0.21 |
| [Soltu.DM.10G000440.1](http://spuddb.uga.edu/cgi-bin/annotation_report.cgi?orf=Soltu.DM.10G000440.1) | 10 | 337 | St2ODD171 | 39223.65 | 5.61 | Extracellular | -0.39 |
| [Soltu.DM.10G011910.1](http://spuddb.uga.edu/cgi-bin/annotation_report.cgi?orf=Soltu.DM.10G011910.1) | 10 | 214 | St2ODD175 | 24903.3 | 8.83 | Extracellular | -0.46 |
| [Soltu.DM.10G007320.2](http://spuddb.uga.edu/cgi-bin/annotation_report.cgi?orf=Soltu.DM.10G007320.2) | 10 | 352 | St2ODD174 | 39834.11 | 6.7 | Extracellular | -0.14 |
| [Soltu.DM.10G007300.1](http://spuddb.uga.edu/cgi-bin/annotation_report.cgi?orf=Soltu.DM.10G007300.1) | 10 | 304 | St2ODD172 | 34710.52 | 5.3 | Cytoplasmic | -0.46 |
| [Soltu.DM.10G024410.1](http://spuddb.uga.edu/cgi-bin/annotation_report.cgi?orf=Soltu.DM.10G024410.1) | 10 | 679 | St2ODD183 | 75525.18 | 5.64 | Cytoplasmic | -0.3 |
| [Soltu.DM.10G019640.2](http://spuddb.uga.edu/cgi-bin/annotation_report.cgi?orf=Soltu.DM.10G019640.2) | 10 | 290 | St2ODD177 | 32873.52 | 5.93 | Cytoplasmic | -0.37 |
| [Soltu.DM.11G025230.1](http://spuddb.uga.edu/cgi-bin/annotation_report.cgi?orf=Soltu.DM.11G025230.1) | 11 | 352 | St2ODD199 | 39236.72 | 5.43 | Extracellular | -0.23 |
| [Soltu.DM.11G025360.1](http://spuddb.uga.edu/cgi-bin/annotation_report.cgi?orf=Soltu.DM.11G025360.1) | 11 | 372 | St2ODD200 | 42566.55 | 6.89 | Extracellular | -0.39 |
| [Soltu.DM.11G025110.1](http://spuddb.uga.edu/cgi-bin/annotation_report.cgi?orf=Soltu.DM.11G025110.1) | 11 | 356 | St2ODD194 | 40564.43 | 5.41 | Cytoplasmic | -0.39 |
| [Soltu.DM.11G025100.1](http://spuddb.uga.edu/cgi-bin/annotation_report.cgi?orf=Soltu.DM.11G025100.1) | 11 | 357 | St2ODD193 | 40494.19 | 5.3 | Cytoplasmic | -0.39 |
| [Soltu.DM.11G025130.1](http://spuddb.uga.edu/cgi-bin/annotation_report.cgi?orf=Soltu.DM.11G025130.1) | 11 | 356 | St2ODD196 | 40485.43 | 5.9 | Cytoplasmic | -0.36 |
| [Soltu.DM.11G025090.1](http://spuddb.uga.edu/cgi-bin/annotation_report.cgi?orf=Soltu.DM.11G025090.1) | 11 | 357 | St2ODD192 | 40220.15 | 5.14 | Extracellular | -0.12 |
| [Soltu.DM.11G004860.1](http://spuddb.uga.edu/cgi-bin/annotation_report.cgi?orf=Soltu.DM.11G004860.1) | 11 | 373 | St2ODD186 | 42429.54 | 6.6 | Cytoplasmic | -0.33 |
| [Soltu.DM.11G010780.1](http://spuddb.uga.edu/cgi-bin/annotation_report.cgi?orf=Soltu.DM.11G010780.1) | 11 | 359 | St2ODD188 | 40485.28 | 5.64 | Cytoplasmic | -0.27 |
| [Soltu.DM.11G009500.1](http://spuddb.uga.edu/cgi-bin/annotation_report.cgi?orf=Soltu.DM.11G009500.1) | 11 | 319 | St2ODD187 | 36316.77 | 5.05 | Cytoplasmic | -0.6 |
| [Soltu.DM.11G025120.1](http://spuddb.uga.edu/cgi-bin/annotation_report.cgi?orf=Soltu.DM.11G025120.1) | 11 | 357 | St2ODD195 | 40378.03 | 5.17 | Cytoplasmic | -0.38 |
| [Soltu.DM.11G003250.1](http://spuddb.uga.edu/cgi-bin/annotation_report.cgi?orf=Soltu.DM.11G003250.1) | 11 | 336 | St2ODD185 | 38436.26 | 6.34 | Cytoplasmic | -0.19 |
| [Soltu.DM.11G017760.1](http://spuddb.uga.edu/cgi-bin/annotation_report.cgi?orf=Soltu.DM.11G017760.1) | 11 | 369 | St2ODD191 | 41075 | 5 | Cytoplasmic | -0.25 |
| [Soltu.DM.11G025160.1](http://spuddb.uga.edu/cgi-bin/annotation_report.cgi?orf=Soltu.DM.11G025160.1) | 11 | 356 | St2ODD198 | 40378.24 | 5.77 | Cytoplasmic | -0.38 |
| [Soltu.DM.11G025140.1](http://spuddb.uga.edu/cgi-bin/annotation_report.cgi?orf=Soltu.DM.11G025140.1) | 11 | 357 | St2ODD197 | 40325.85 | 5.37 | Cytoplasmic | -0.41 |
| [Soltu.DM.11G016970.1](http://spuddb.uga.edu/cgi-bin/annotation_report.cgi?orf=Soltu.DM.11G016970.1) | 11 | 357 | St2ODD190 | 40671.34 | 5.7 | Cytoplasmic | -0.38 |
| [Soltu.DM.11G010950.1](http://spuddb.uga.edu/cgi-bin/annotation_report.cgi?orf=Soltu.DM.11G010950.1) | 11 | 352 | St2ODD189 | 40040.81 | 5.6 | Cytoplasmic | -0.43 |
| [Soltu.DM.12G000090.1](http://spuddb.uga.edu/cgi-bin/annotation_report.cgi?orf=Soltu.DM.12G000090.1) | 12 | 354 | St2ODD201 | 40148.03 | 5.85 | Cytoplasmic | -0.31 |
| [Soltu.DM.12G023340.1](http://spuddb.uga.edu/cgi-bin/annotation_report.cgi?orf=Soltu.DM.12G023340.1) | 12 | 316 | St2ODD203 | 35998.29 | 5.32 | Cytoplasmic | -0.4 |
| [Soltu.DM.12G010750.2](http://spuddb.uga.edu/cgi-bin/annotation_report.cgi?orf=Soltu.DM.12G010750.2) | 12 | 315 | St2ODD202 | 36247.68 | 6.47 | Extracellular | -0.4 |
| [Soltu.DM.12G023970.2](http://spuddb.uga.edu/cgi-bin/annotation_report.cgi?orf=Soltu.DM.12G023970.2) | 12 | 293 | St2ODD205 | 33012.75 | 5.49 | Cytoplasmic | -0.24 |
| [Soltu.DM.12G023970.1](http://spuddb.uga.edu/cgi-bin/annotation_report.cgi?orf=Soltu.DM.12G023970.1) | 12 | 364 | St2ODD204 | 41091.92 | 6.08 | Cytoplasmic | -0.32 |
