# Supplementary material for: Genome-wide identification of 2-oxoglutarate and Fe (II)-dependent dioxygenase family genes and their expression profiling under drought and salt stress in potato
Source: PeerJ. 2023 Nov 20;11:e16449. doi: 10.7717/peerj.16449 (PMC10666615; doi:10.7717/peerj.16449)
Supplement: Supplemental Information 3 [file peerj-11-16449-s003.docx]

| **Gene** | **Class** | **Center_X** | **Center_Y** | **Center_Z** | **Binding affinity (Kcal/mol)** |
| --- | --- | --- | --- | --- | --- |
| St2ODD29 | GaOx | 3.90 | -4.03 | -9.52 | -3.9 |
| St2ODD124 | GaOx | 6.69 | -6.15 | -9.40 | -7.7 |
| St2ODD85 | FNS | 64.72 | 20.32 | -23.18 | -6.9 |
| St2ODD87 | FNS | 2.88 | -4.20 | -9.39 | -8.5 |
| St2ODD118 | ACOs | -9.79 | -0.83 | 7.70 | -7 |
| St2ODD120 | ACOs | 32.85 | 45.11 | 35.27 | -8 |
