# Supplementary material for: Genome-wide identification of 2-oxoglutarate and Fe (II)-dependent dioxygenase family genes and their expression profiling under drought and salt stress in potato
Source: PeerJ. 2023 Nov 20;11:e16449. doi: 10.7717/peerj.16449 (PMC10666615; doi:10.7717/peerj.16449)
Supplement: Supplemental Information 4 [file peerj-11-16449-s004.docx]

| **Gene** | **Forward primer sequences (5'-3')** | **Reverse primer sequences (5'-3')** |
| --- | --- | --- |
| EF1α | ACCAAGATTGACAGGCGTTC | CCTTCTTCTCCACAGCCTTG |
| St2ODD34 | ACACAAGTGGCCTCCAAATC | CACCCTGGCTTTCAAACAAT |
| St2ODD54 | AAAGTGGTGGGTGTCTGTCA | TGGAACTGATGCCAAGGAGT |
| St2ODD25 | AGGTCTCCAGGTTCAGCATC | CATCAATGGAGAAGCTGGCC |
| St2ODD130 | CACTCTCCTAGCAAACCCGA | CAAGTGCAAGTTCTGGCTGA |
| St2ODD22 | TTTCCCTTAGTGTGGCGGAG | TCACAATGAGGTCCTGTCCC |
| St2ODD112 | AGCAAGTCTGTTCAGTGTGC | ATGGACGAAATTTGCAGGCA |
| St2ODD138 | CTGCCACCACAGCTCCTATA | CCTTGAGGTGGTTTGGTTCG |
| St2ODD76 | TACTCCAAGCTTTCGCGATT | CCCATTCAGAAGAAGCCTTG |
| St2ODD91 | TGGTTGTTCAGAGGGGTTTC | ACCGCTTGCCAGTACTCTGT |

**Quantitative primers of the selected enzyme-encoding genes specific for drought and heat stress**
