# Supplementary material for: Genome-wide identification of 2-oxoglutarate and Fe (II)-dependent dioxygenase family genes and their expression profiling under drought and salt stress in potato
Source: PeerJ. 2023 Nov 20;11:e16449. doi: 10.7717/peerj.16449 (PMC10666615; doi:10.7717/peerj.16449)
Supplement: Supplemental Information 5 [file peerj-11-16449-s005.docx]

| **Motifs** | **Sequence** |
| --- | --- |
| Motif 1 | GQSLRVNYYYPPCPPELTGGTPHHDDPGLLTLLQQBQVGGLQVLKDGQWIDVPPJPNALVVNIGDLLZILSNGRYKSVEHRVVVNSEKERISVA |
| Motif 2 | IVEKIREACEEWGFFQVINHGIPSELLEKMKDGTKEFFELPLEEKKKYYSSDPTKEGVGQSFFDLFSEKLDWWDDFTLSTLP |
| Motif 3 | NPPLWPELPPSFREVVEEYSKEVKKLGKKJLELLSEALGLEENHLKDMFC |
| Motif 4 | FFLNPKLDKVIGPAKELVDEENPPLYKPFTVSDYLKYFFSKGLDGKSSLD |
| Motif 5 | LKAFDDTKAGVKGLVDSGIVKIPRIFIQPPEERPEKSQINKSESIPPIDD |
| Motif 6 | AGGQGMRVNYYPPCPQPELTLGLSPHSDPDFLTILLQDNIGGLQQLHKBQWWDVPPPPGALVVNIGDDLQ |
| Motif 7 | KVKSFDSSSKKKESNIPSQYIPPDZEKSPAVSQELSVPVIDLQGLISGDP |
| Motif 8 | ZVPDLDDEYREVMKDFAKRLEKLAEELLDLLCENLGLEKGYLKKAFYGSK |
| Motif 9 | TAPYSVPPVEIKLLKESPELRLIPENYVGPYICRSGDDADEKEDIMDSIP |
| Motif 10 | QLINHGVSPSVVEKMKHETQKFFDLPLEEKKKFEQSEGDTDGFGQLFVVSEEQKLDWADLFYJKTAPPYJ |
